# From palm to practice: prescription digital therapeutics for mental and brain health at the National Institutes of Health

**DOI:** 10.3389/fpsyt.2024.1433438

**Published:** 2024-09-10

**Authors:** Michele Ferrante, Layla E. Esposito, Luke E. Stoeckel

**Affiliations:** ^1^ Division of Translational Research, National Institute of Mental Health, Bethesda, MD, United States; ^2^ Division of Behavioral and Social Research, Eunice Kennedy Shriver National Institute of Child Health and Human Development, Bethesda, MD, United States; ^3^ Division of Extramural Research, National Institute on Aging, Bethesda, MD, United States

**Keywords:** prescription digital therapeutics (PDT), software as a medical device (SaMD), digital mental health assessment and interventions, aging adults, youth & adolescence, digital psychiatry, computational psychiatry, Digital Health Technologies (DHTs)

## Abstract

Prescription Digital Therapeutics (PDTs) are emerging as promising tools for treating and managing mental and brain health conditions within the context of daily life. This commentary distinguishes PDTs from other Software as Medical Devices (SaMD) and explores their integration into mental and brain health treatments. We focus on research programs and support from the National Institutes of Health (NIH), discussing PDT research supported by the NIH’s National Institute on Child Health and Development (NICHD), National Institute of Mental Health (NIMH), and National Institute on Aging (NIA). We present a hierarchical natural language processing topic analysis of NIH-funded digital therapeutics research projects. We delineate the PDT landscape across different mental and brain health disorders while highlighting opportunities and challenges. Additionally, we discuss the research foundation for PDTs, the unique therapeutic approaches they employ, and potential strategies to improve their validity, reliability, safety, and effectiveness. Finally, we address the research and collaborations necessary to propel the field forward, ultimately enhancing patient care through innovative digital health solutions.

## Introduction

Digital Health Technologies (DHTs), such as mobile apps, telemedicine, and wearables, are transforming healthcare delivery and management. Software as a Medical Device (SaMD) (1) is a subset of DHTs used for medical purposes like diagnosis, treatment, and prevention. Software in a Medical Device (SiMD), on the other hand, is software embedded within a physical medical device, powering its mechanics, or processing its information (e.g., software for neuromodulation, automatic drug delivery, and brain imaging). SaMDs are also distinct from wellness applications that promote a healthy lifestyle (e.g., physical and cognitive activity, sleep, mindfulness-based stress reduction), but this distinction can sometimes be unclear (1).

**Figure 1:**
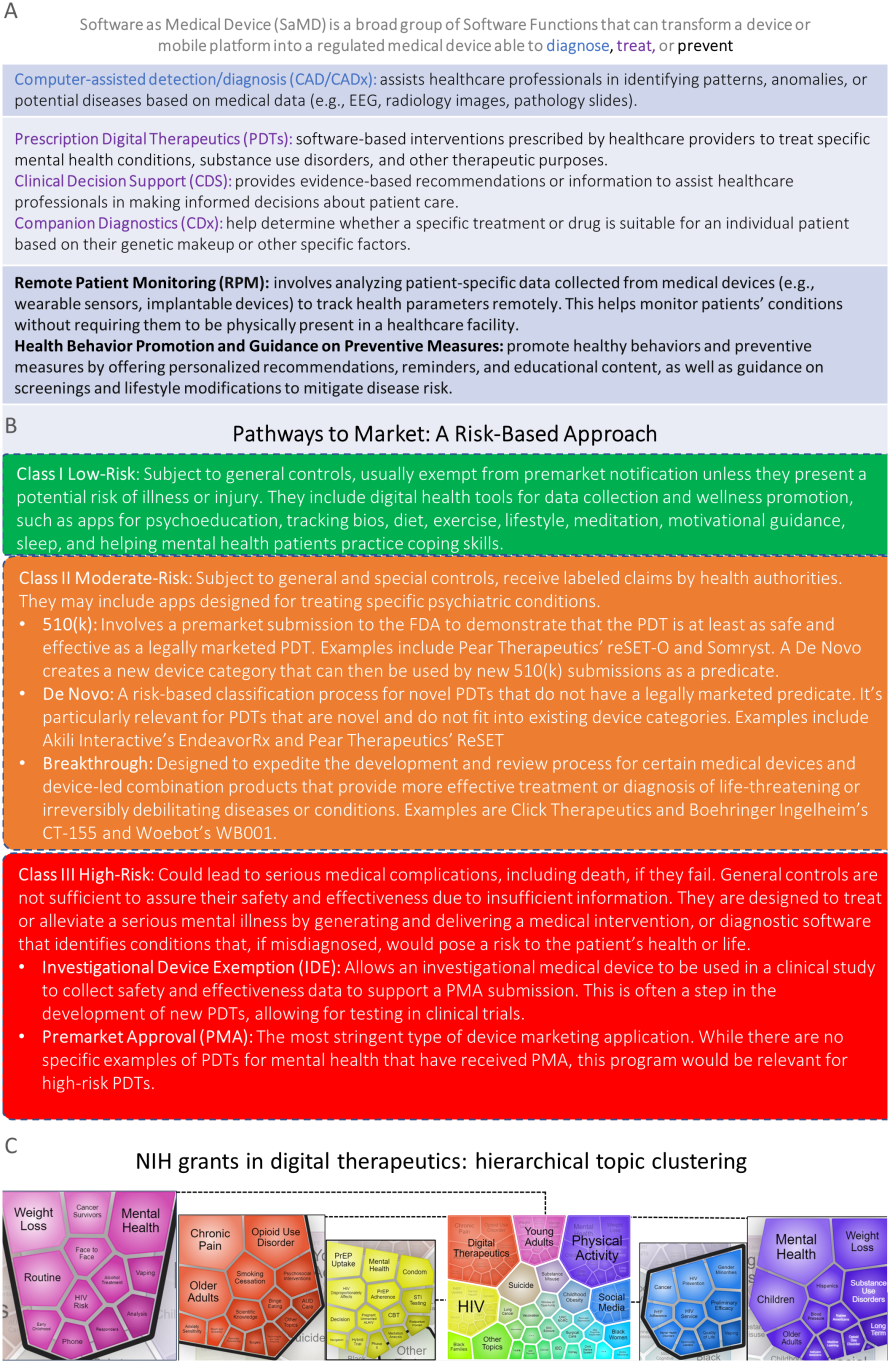
**(A)** Top, Software as Medical Device functions may have different medical purposes (e.g., diagnose, treat, and prevent). **(B)** PDTs: Pathways to Market a Risk-based Approach. Federal Food, Drug, and Cosmetic Act, section 513, established the risk-based device classification system for medical devices that also applies to PDTs. Each device is assigned to one of three regulatory classes: Class I (top, green), Class II (middle, orange), or Class III (bottom, red), based on the level of control necessary to provide reasonable assurance of its safety and effectiveness. There are several distinct pathways to market for PDTs such as IDEs, PMAs, 510(k), *De Novo*, and breakthrough devices. PDTs require clinical evidence, regulatory oversight, prescription, and FDA clearance to ensure safety and efficacy. **(C)** Unofficial Data: Hierarchical topic clusters were identified through natural language processing analyses of all NIH active grants in digital therapeutics. A total of 138 grants were classified as digital therapeutics if they contained the terms “digital therapeutics”, “digital health intervention”, “digital intervention”, “behavioral health software”, or “software-based therapeutics” in their titles, specific aims, or summaries.

SaMD includes prescription digital therapeutics (PDTs), clinical decision support, companion diagnostics, computer-assisted detection/diagnostics, remote monitoring, and multifunctional devices (**Figure 1A**). In the United States, SaMD has distinct regulatory pathways to market based on factors like risk level (**Figure 1B**) and intended purpose (2). PDTs are a U.S. Food and Drug Administration (FDA)-regulated SaMD for treating patients that are altering the landscape of behavioral health diagnostics and care (3, 4).

PDTs share aspects with pharmacotherapies, including development cycles, clinical trials, and regulatory review (3–5). PDTs can be used alone or with other treatments to optimize therapeutic interventions across demographics, conditions, and contexts. PDTs may offer advantages over pharmacotherapy (e.g., real-time testing and intervention, closed-loop or adaptative designs for optimization, tailoring personalized interventions, flexible regulatory authorizations, reduced time to market, and benign side effect profiles) making them promising mental and brain health intervention tools (3).

There are also challenges developing and evaluating rigorous, evidence-based, high-quality PDTs (6), including, but not limited to, addressing usability and accessibility barriers (7) and involving communities and populations that use these tools in the co-development process (8). There is also a need to develop technical and medical standards (9) and regulatory science tools (10) for mental and brain health PDTs to ensure safety, efficacy, and widespread adoption of mental and brain health PDTs by providing a structured framework that supports innovation while maintaining high standards of care and patient safety (11).

## The role of the National Institutes of Health (NIH)

The NIH supports research on DHTs in mental and brain health (12). The Science of Behavior Change (SOBC) program advances behavioral intervention development and transparency (13–16) by strengthening rigor and advancing a mechanistic framework for behavior change research at every stage of intervention development. The NIH Stage Model (17) offers guidance on best practices for generating, testing, and implementing effective mechanisms-focused behavioral interventions that can be delivered in real-world settings for regulatory agency consideration (18). This includes PDTs as supplementary or alternative tools for treatment, accessible beyond traditional healthcare settings (18).

For instance, the National Institute on Drug Abuse (NIDA) supported the Center for Technology and Behavioral Health (CTBH) (19), focusing on substance use and co-occurring disorders. CTBH’s interdisciplinary work spans treatment development, evaluation, emerging technologies, data analytics, dissemination, and implementation, leveraging a diverse team with expertise in various fields.

Foundational work supported by NIH, including programs like SOBC and CTBH, led to the first FDA authorizations for PDTs, namely reSET^®^ and reSET-O^®^ (Pear Therapeutics) (20). However, the Institute for Clinical and Economic Review (ICER) expressed concerns about the quality of evidence supporting DHTs like reSET-O^®^, citing potential health, financial, and moral risks (21).

Pear Therapeutics, the company behind reSET^®^ and reSET-O^®^, filed for bankruptcy after failing to secure broad insurance coverage for their products (22). It is suggested that additional, rigorous studies and trials, including real-world evidence, may be necessary to demonstrate that PDTs will save costs in the long term.

**Figure 2:**
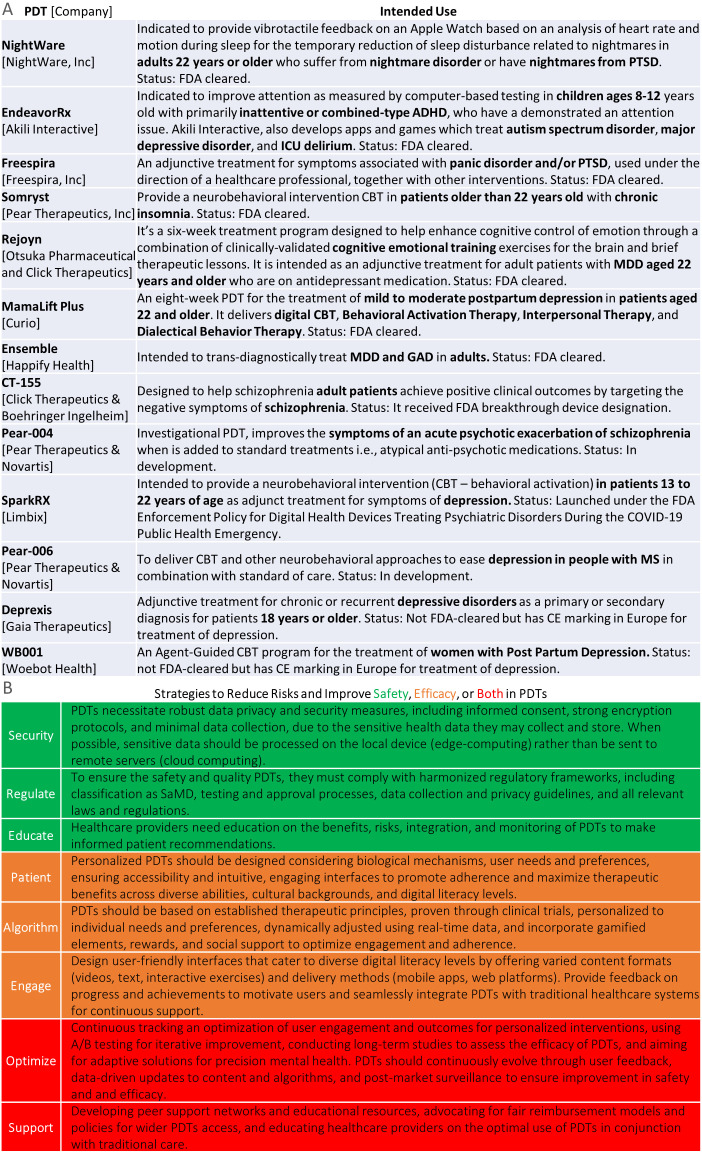
Top, examples of PDTs across different mental health disorders and age groups. Bottom, strategies to improve safety and effectiveness in PDTs.

**Figure 2A** shows examples of PDTs intended for various conditions. For instance, Rejoyn (23) is authorized for adult depression and includes cognitive training, specifically the Emotional Faces Memory Task (EFMT), an adaptive emotional working memory task. While Cognitive Behavioral Therapy (CBT) is well-established, EFMT is less so (24). This underscores the need for external validation to determine if cognitive training improvements impact daily activities, quality of life, and real-world functional outcomes (25, 26). On this note, **Figure 2B** presents eight potential strategies to improve safety and effectiveness in PDTs.

## The National Institute of Child Health and Development (NICHD)

NICHD funds research under the Children and Media Research Advancement Act (CAMRA), signed into law in 2022 (27), authorizing NIH to study the effects of technology and digital media on youth (28). This is crucial for PDT development, with research investments growing from $3.4 million in 2020 to an estimated $16.8 million in 2023.

Young individuals are often considered “digital natives” (29) due to their ubiquitous access to technology (e.g., computers, internet, smartphones) integrated into their daily lives for education, communication, and entertainment since birth. Device interaction frequently involves multitasking and switching between multiple digital devices and platforms, such as smartphones and computers, often simultaneously (29).

In education, digital technology is often a core feature, including retrieving information from the Internet and personalized, visual, and interactive forms of engagement and instruction. Social interactions often occur online through social media, messaging apps, and multiplayer games, marking a significant shift from previous generations’ behavioral norms and cognitive processes. Social media has emerged as a cost-effective tool to enhance patient engagement and recruitment for trials, but it also raises ethical, legal, and social implications (12, 30).

For mental health, we need more effective, evidence-based PDTs to help youth with conditions like anxiety, depression, ADHD, disruptive behavior disorders, and eating disorders. Building evidence for technology use to address unmet mental health needs and identify and mitigate harms is critical. User-friendly interfaces, gamification, and virtual reality (VR) could make patients more engaged. PDTs enabled by AI/ML could help reduce or amplify disparities (31) in mental and behavioral health care. They could enable remote personalization, monitoring, and feedback, allowing healthcare providers to track progress and adjust (12). They could also tailor interventions to individual needs, tracking real-time progress and feedback by healthcare providers while overcoming the limitations of self-reporting (32). PDTs could also help capture contextual and environmental data influencing children’s health outcomes (33–35).

Additionally, accounting for differences in DHTs literacy among children and caregivers, cultural and linguistic appropriateness, and clear data privacy information and safeguards is crucial. User-friendly interfaces with gamification, interactivity, or VR devices could boost youth engagement and lower treatment resistance.

Given the sparing use of psychotropic medications in pediatric populations due to potential side effects, time, cost, and accessibility issues, PDTs could serve as a logical solution, addressing these challenges by providing evidence-based treatments for mental and brain health conditions (5) in youth, including those with intellectual and developmental disabilities such as Down syndrome (36, 37).

Looking to the future, NIH has encouraged research on the impact of technology, digital, and social media use on children and adolescents’ development and mental health (38, 39). NICHD and NIMH held a workshop on these topics (40). NICHD is building a research agenda to better assess the effects of technology and digital media on the developmental trajectories and mental health outcomes of infants, children, and adolescents.

## The National Institute of Mental Health (NIMH)

NIMH focuses on applying computational solutions transdiagnostically and to a wide range of mental health conditions, including schizophrenia, bipolar disorder, depression, anxiety disorders, autism spectrum disorders, eating disorders, PTSD, OCD, ADHD, borderline personality disorder, panic disorder, social anxiety disorder, and generalized anxiety disorder.

NIMH also aims to develop computational solutions to address mental health issues in children, families, minority groups, and elders. By supporting innovative research and funding studies on digital interventions, NIMH aims to identify evidence-based solutions that can be widely implemented to improve mental health outcomes in diverse populations. DHTs, such as telehealth services and PDTs, offer the potential to reach individuals regardless of their location, providing personalized and effective care. While acknowledging the challenges related to privacy, data security, and rigorous evaluation, NIMH is committed to addressing these issues to maximize the potential of PDTs. Ultimately, NIMH sees digital mental health as a key component of modern mental health care.

For this reason, NIMH has adopted a comprehensive strategy to advance research in DHTs and PDTs, identifying both the opportunities and challenges implicit in this evolving field (41, 42). These initiatives laid the groundwork for several significant efforts aimed at using computational methods to improve mental health care.

A prominent NIMH’s initiative includes the development of personalized tools specifically designed for treatment assignment in depression (43) with suicide being an area of high priority. Furthermore, the IMPACT-MH projects utilize computationally-informed behavioral phenotypes, enhancing our ability to predict individual outcomes in various mental disorders (44). NIMH encourages the use of computational phenotyping and longitudinal dynamics to inform clinical decision-making in psychiatry (45). Additionally, the institute is committed to supporting small businesses that are engaged in developing, evaluating, and commercializing DHTs within high-priority areas (46).

Moreover, the Research Domain Criteria Initiative (RDoC) has introduced gamified tasks that aid in studying mental disorders, with large-scale efforts such as the ‘All of Us’ project facilitating in-depth phenotyping (47, 48). In tandem with these efforts, NIMH has recently launched specialized laboratories to optimize DHTs (49) and undertake other efforts aimed at both optimizing and validating these technologies (50). Validation of dimensional constructs pertinent to psychopathology is another key focus for NIMH (51), along with promoting the use of advanced computational methods in RDoC research (52).

NIMH’s collaboration with the National Science Foundation (NSF) on the Smart Health and Biomedical Research program further exemplifies its commitment to integrating technology and health (53). In addition, through the Computationally-Defined Behaviors Initiative, detailed behavioral assays have been developed (54). On a broader scale, NIMH’s mobile health initiative seeks to improve outcomes in low- and middle-income countries through technological innovations (55), while the Health IT initiative is dedicated to reducing healthcare disparities (56). The institute has also made strides in developing explainable AI and machine learning methods to enhance mental health research (57, 58).

Furthermore, NIMH is focused on creating data archives, informatics tools, and data standards that are crucial for brain-behavior research (59). Additionally, the institute emphasizes the importance of early interventions for psychosis (60, 61) and seeks to identify novel therapeutic targets and biomarkers in schizophrenia (62, 63), ensuring that all generated data and analyses are readily accessible.

Collaborations between NIMH, the Office of Data Science Strategy (ODSS), and the Office of Behavioral and Social Sciences Research (OBSSR) could greatly advance the development of PDTs. By leveraging NIMH’s mental health innovations, ODSS’s data-driven methods, and OBSSR’s behavioral and social insights, they can create integrated ecosystems for effective and personalized PDTs. This partnership could aim to make evidence-based PDTs, more innovative, scalable, and accessible to diverse populations, transforming mental health care delivery. For instance, by leveraging AI and cognitive computing technologies (64), one could envision a future where PDTs can target transdiagnostic constructs, such as reframing negative thoughts, building social confidence, enhancing emotional regulation, and fostering virtual social connections. This holistic approach aspires to significantly impact those grappling with loneliness, social isolation, and other critical aspects of various mental health conditions (65).

## The National Institute on Aging (NIA)

NIA leads research on DHTs, including AI/ML, to improve health outcomes for older adults. Today’s older adults are not digital natives and may be slower to adopt DHTs. There are age-related changes in neuropsychological functions (66, 67) such as, slowed information processing (68) and a preference for information accuracy over volume or speed (66–69), which may make the often fast-paced, continuously changing digital landscape more challenging to manage or a less preferred environment for some older adults. As people age, their socio-emotional functioning become more predictable and consistent, with fewer negative emotions. Social networks become less expansive with a shift from exploring and building larger social networks to a focus on fewer, closer relationships (72). As many older adults did not develop in an era of pervasive digital technology, many may not have developed mental models of digital tool functionality (70). However, these technologies could be co-developed with users to increase usability and accessibility. These technologies can address issues like limited mobility, cognitive aging, and social isolation. Sensors in devices can monitor behavior in the living environment, including the home, offering new strategies for assistive care (71). Tools for remote monitoring and supportive care can be particularly beneficial for adaptive aging. The Collaborative Aging Research Using Technology (CART) initiative uses sensor technology and analytics to assess activity in older adults’ homes and detect meaningful changes longitudinally (73). Technologies like those used in CART or Emerald (passive radio-wave-based home monitor) (71), including wall and ceiling sensors, door sensors, digital scales, digital pillboxes, smartwatches, and driving sensors, offer the potential to detect changes in health indicators in the free-living environment where most older adults prefer to function independently (12).

NIA’s digital health initiatives include mobile health (mHealth), sensing technologies, wearables, telemedicine, and adaptive behavioral interventions. This includes AI-based projects for diagnostics, caregiving, and digital therapeutics, such as online therapy and cognitive impairment screening. The AITC program fosters AI technology development, pilot studies, and best practices for integrating AI in healthcare for older adults, collaborating with private industry, venture capital firms, and healthcare systems to drive innovation (74). NIA’s Small Business Programs provide early-stage funding for AI and ML innovations in diagnostics, patient monitoring, and other digital health solutions, fostering entrepreneurial endeavors in aging research (75).

NIA leads research on Alzheimer’s Disease and related dementias (AD/ADRD), aiming to prevent these diseases, improve cognitive and brain health, reduce caregiver and economic burdens, and address health disparities. NIA develops digital tools for early detection of subtle changes in cognition, emotion, social behavior, and daily functions, which can inform diagnostics, treatments, and important life decisions (76, 77). This also includes use for prevention (78) and treatment of AD/ADRD (79) targeting many of the modifiable risk factors for AD/ADRD. One example is cognitive training. NIA supports the Preventing Alzheimer’s with Cognitive Training (PACT) trial (80) and the Adaptive Clinical Trial of Cognitive Training to Improve Function and Delay Dementia: The ACTIVE MIND Trial (81) to test the components in cognitive training interventions that may prevent AD/ADRD and improve daily function in individuals with early changes due to AD/ADRD such as Mild Cognitive Impairment. NIA will review the evidence supporting cognitive training and identify research gaps and opportunities to advance the field in an upcoming Cognitive Training Webinar Series (82). PDTs have the potential to update cognitive training intervention approaches through novel design features like closed-loop, adaptive designs, gamification, and augmented reality (83). NIA’s role in PDT research aims to address the unique challenges of aging, promoting healthier and more independent living for older adults.

These NIH-led efforts have significantly contributed to the development of digital therapeutics as an active area of research. A total of 138 NIH grants were categorized as digital therapeutics if they included the words “digital therapeutics”, “digital health intervention”, “digital intervention”, “behavioral health software”, or “software-based therapeutics” in their titles, specific aims, or summaries. We used the iSearch natural language processing visualization function to identify 29 distinct hierarchical topic clusters from all the currently active NIH grants related to digital therapeutics. For the five largest topics -digital therapeutics, physical activity, HIV, young adults, and social media- we plotted their respective subtopics to provide a clearer understanding of the main areas covered by NIH-funded research in digital therapeutics (**Figure 1C**). Several emerging topics for instance, social media, young adults, older adults, opioid use disorders, mental health, black women/families, Hispanics, Native Americans, CBT, and suicide map very well with NIH areas of high priority discussed above.

## Discussion

PDTs are innovative tools for managing mental and brain health, distinct from other SaMDs due to their unique regulatory frameworks. They offer advantages over traditional pharmacotherapies, such as real-time testing and fewer side effects, and can be used independently or alongside other treatments. Successful PDT development requires attention to usability, accessibility, clinical workflow, and regulatory standards, with community involvement and strong clinical evidence being key for adoption. Most PDTs are rooted in behavioral science concepts from the 1950s and 60s, particularly CBT. Newer methods integrate neuroscience and precision psychiatry, leveraging insights from various fields (84–92).

To maximize effectiveness, PDTs should focus on cognitive health equity, co-developing with low-access populations, and employing mechanisms-focused frameworks like the NIH Stage Model (17). The NIH supports PDT research by addressing current issues such as technology’s impact on youth (40), social isolation, and tools for age-related cognitive decline and disease prevention, including Alzheimer’s.

However, despite their potential, PDTs face critical challenges. One concern is the variability in their clinical efficacy, as some PDTs may not deliver consistent results across diverse populations, conditions, or biotypes, leading to questions about their effectiveness compared to traditional therapies. Additionally, the dependence on technology raises concerns about accessibility; individuals in low-income or rural areas may lack the necessary devices or internet connectivity, exacerbating existing health disparities. Furthermore, regulatory and reimbursement frameworks for PDTs are still evolving, which can hinder their integration into conventional healthcare settings—leading to uncertainty among providers about prescribing them. The reliance on self-reported data also poses issues, as it can be influenced by user bias and motivation. Therefore, while PDTs represent a promising advancement in mental health care, careful consideration of these limitations and ongoing evaluation of their impact is essential for their successful implementation.

## Areas of need

- Adaptive, Mechanistic Intervention Development: Emphasizing just-in-time approaches and optimizing personalized interventions using systems science principles (93). Frameworks like experiment-in-a-box align with NIH’s mechanisms-focused strategies (94).
- Inclusive Digital Measurements: DHTs enhance clinical trials through real-world data collection, improving engagement and participation in under-represented populations (12, 95).
- AI-Driven Metrics: Utilizing AI to identify patterns and improve diagnosis, treatment, and prognosing while addressing interpretability and clinical validity challenges in real-world applications (58, 96, 97).

AI/ML-enabled PDTs leverage sensors to create data-driven treatment plans for various age groups, enhancing outcomes and patient insights. Ensuring different types of validity is essential for their reliability and efficacy (98–100). The NIH is interested in expanding PDT research, especially in validating DHTs (50) in underrepresented populations.

Challenges for low-access communities include limited internet access, device availability, and digital literacy. Regulatory hurdles arise from rapid technological advancements (3, 9, 10), with agencies needing to navigate increased health app complexities. Addressing these issues could transform mental health care, expanding access and improving engagement while ensuring privacy and ethical standards are met. PDT standardization and interoperability are vital for collaboration and data sharing that may enable democratization (101).

DHT implementation calls for creative strategies and stakeholder training, with robust study designs necessary for effectiveness assessment. User-centered co-design is crucial for long-lasting innovations, and standardized reporting guidelines will enhance care quality.

In summary, while PDTs present significant promise for more personalized and effective mental health treatments, challenges regarding validity, privacy, and infrastructure must be addressed. Future research should focus on identifying transdiagnostic mechanisms and developing products that utilize this knowledge effectively.

## Data Availability

We are unable to share the data due to its sensitive and identifiable nature, as it pertains to specific NIH funded grants. Further inquiries can be directed to the corresponding author. Requests to access the datasets should be directed to MF, michele.ferrante@nih.gov.
